# IL-12 and GM-CSF in DNA/MVA Immunizations against HIV-1 CRF12_BF Nef Induced T-Cell Responses With an Enhanced Magnitude, Breadth and Quality

**DOI:** 10.1371/journal.pone.0037801

**Published:** 2012-05-24

**Authors:** Ana María Rodríguez, María Fernanda Pascutti, Cynthia Maeto, Juliana Falivene, María Pía Holgado, Gabriela Turk, María Magdalena Gherardi

**Affiliations:** Centro Nacional de Referencia para el SIDA, Universidad de Buenos Aires, Buenos Aires, Argentina; University of Colorado Denver, United States of America

## Abstract

In Argentina, the HIV epidemic is characterized by the co-circulation of subtype B and BF recombinant viral variants. Nef is an HIV protein highly variable among subtypes, making it a good tool to study the impact of HIV variability in the vaccine design setting. We have previously reported a specific cellular response against NefBF with low cross-reactivity to NefB in mice. The aim of this work was to analyze whether the co-administration of IL-12 and GM-CSF, using DNA and MVA vaccine vectors, could improve the final cellular response induced. Mice received three DNA priming doses of a plasmid that express NefBF plus DNAs expressing IL-12 and/or GM-CSF. Afterwards, all the groups were boosted with a MVAnefBF dose. The highest increase in the magnitude of the NefBF response, compared to that induced in the control was found in the IL-12 group. Importantly, a response with higher breadth was detected in groups which received IL-12 or GM-CSF, evidenced as an increased frequency of recognition of homologous (BF) and heterologous (B) Nef peptides, as well as a higher number of other Nef peptide pools representing different viral subtypes. However, these improvements were lost when both DNA cytokines were simultaneously administered, as the response was focused against the immunodominant peptide with a detrimental response towards subdominant epitopes. The pattern of cytokines secreted and the specific-T-cell proliferative capacity were improved in IL-12 and IL-12+GM-CSF groups. Importantly IL-12 generated a significant higher T-cell avidity against a B heterologous peptide.

This study indicates that the incorporation of DNA expressing IL-12 in DNA/MVA schemes produced the best results in terms of improvements of T-cell-response key properties such as breadth, cross-reactivity and quality (avidity and pattern of cytokines secreted). These relevant results contribute to the design of strategies aimed to induce T-cell responses against HIV antigens with higher quality.

## Introduction

Thirty years has passed from the first reported cases of aids and, despite the health community efforts, the epidemic has not been stopped yet. The last report from the Joint United Nations Program on HIV/aids (UNAIDS) estimated nearly 34 million people [31.6 million–35.2 million] living with HIV at the end of 2010, while the new infections were estimated in 2.7 million [2.4 million–2.9 million] people [1]. HIV/aids is one of the major health issues in developing countries, where it is difficult to access to antiretroviral therapy. In these countries, only a few fraction of the population in need of therapy has access to it. Undoubtedly the most effective measure to combat the epidemic will be the possibility to apply a safe and effective vaccine.

One of the challenges to solve in the HIV vaccine development, is the high variability of the virus, which difficult the antigen selection. The M group of HIV-1, responsible for the pandemic, have been differentiated in ten subtypes (A–K) and sub-subtypes (A1–A4 and F1–F2) [2]. Moreover, the complexity has been elevated with the dissemination of 51 circulating recombinant forms (CRF) with a defined genetic structure.

In Argentina, the number of HIV^+^ people is estimated in 130.000 [3]. There are many studies on the HIV molecular epidemiology in different Argentinean populations, where the presence of B subtype and BF recombinant forms were described, with a high prevalence of CRF12_BF variant [4], [5], [6]. These recombinant forms were reported in other South American countries such as Uruguay, Brazil and Ecuador, among others [4], [7], [8], [9]. Also, the relevance of subtype-specific immune responses have not been totally defined [10], [11], [12], imposing the necessity of considering the regional epidemic molecular patterns on the vaccine designs.

Nef is an HIV protein used in different vaccine development strategies [http://www.avac.org/ht/a/GetDocumentAction/i/3436] [13], [14], [15], [16]. A feature that makes Nef a good vaccine antigen candidate is its early expression in the viral life cycle [17], making it a relevant target during the early steps of cell infection. Furthermore, several reports show the early immunogenicity of Nef [18], [19], [20]. Addo and coworkers studied HIV patients in different infection stages and they observed that Nef and Gag were the most recognized HIV proteins containining the highest density of epitopes. They also demonstrated that the two most frequently recognized peptides were from Nef [19]. On the other hand, Nef and Env are the most variable HIV proteins, making them good tools to study the impact of HIV variability on the immune cross-recognition (amino acid difference between NefBF and NefB 24.5%).

During the last years, aids vaccine designs were focused on those strategies able to generate a cellular immune response. This was due to the evidences indicating that immune responses mediated by cytotoxic T lymphocyte (CD8^+^ T cells, CTL) played a central role controlling HIV infection and progression to aids [21], [22]. In this sense, different findings from the basic area of vaccine research suggest that three characteristics of the specific CD8^+^ T-cells could be involved in the control of the viremia: frequency, breadth of the epitopes recognized and cell functional quality [23], [24].

The Thailand phase III vaccine clinical trial consisted in four doses of a canarypox viral vector (ALVAC-HIV) expressing Gag and Pol (from subtype B, LAI) and Env (gp120 from CRF01_AE, subtype E, linked to the transmembrane anchoring portion of gp41, subtype B (LAI)), followed by two doses of a recombinant bivalent Env protein AIDSVAX from subtypes E and subtype B (MN). This was the first trial that demonstrated a modest protection, exhibiting a vaccine effectiveness of 31.2%. However, there were no differences in the viral load and CD4^+^ T-cell counts of people who became infected, whether they had received the vaccine or placebo [25]. Importantly, in the Thailand trial, the antigens used in the vaccine were matched to the prevalent subtypes in the region where the vaccine was tested. The results from the Thailand trial represented a breakthrough in the road towards an HIV vaccine, suggesting that it will be important to improve the vaccine-induced immune responses, for instance by incorporating adjuvants during the immunization regimes or by improving the vectors to be used. Even more, due to the high HIV variability, it is critical to explore immunization strategies that promote the generation of T cell responses with a broad reactivity, being capable to recognize a wide spectrum of divergent viral sequences.

Currently, one of the most commonly used strategies in the HIV vaccine field is the prime/boost regimen, administering DNA vectors at prime and viral vectors at boost [26]. For this strategy, some of the most widely employed viral vectors belong to the Poxviridae family, including vaccinia virus in its attenuated variants MVA (modified vaccinia virus Ankara) [27], canarypox (used in the test Thailand phase III [25]) and fowlpox based vectors [28], [29]. Given the limited immunogenicity of the DNA vaccines, an area that has gained importance is the design of “rational” adjuvants is the used of cytokines or chemokines to increase the immunogenicity of expressed antigens. Among these molecules, the most studied include IL-12 (Interleukin-12) [30], GM-CSF (colony-stimulating factor granulocyte-macrophage) [31], IL-18 [32] and IL-15 [33]. It is considered that cytokines would be good candidates to enhance immune responses due to the central role they play in the modulation of the immune system, controlling different aspects of the immune response [34]. For instance, some cytokines have a dominant influence on the polarization of CD4^+^ T-cells toward different T-helper phenotypes [34], [35]. Dendritic cells (DCs) play a crucial role in adaptive immunity by their ability to activate naïve T-cells and to direct the differentiation and polarization of T-cells. By producing IL-12, among other cytokines, mature DCs promote the differentiation of CD4^+^ T lymphocytes into IFN-γ-producing Th_1_ cells, as well as the differentiation of CD8^+^ T lymphocytes into cytotoxic cells [35]. Moreover, some studies suggest that IL-12 and IFN type I could be considered as a signal 3 in the activation and differentiation of CD8^+^ T-cells. This signal could replace classical adjuvants in vaccine formulation [36]. Another important modulator cytokine is GM-CSF which acts on monocyte/macrophages and all granulocytes. GM-CSF also controls DCs and T-cell function, thus linking innate and adaptive immunity [37]. Moreover, it is able to recruit professional antigen presenting cells to the vaccine site administration and to enhance their ability to take up foreign antigens [38]. It has been shown that IL-12 and GM-CSF are able to increase the immune response against hepatitis B virus, Leishmania and HIV antigens, among others [31], [32], [39], and synergistic effects between these two adjuvants were also described [40], [41].

We have previously characterized NefBF immunogenicity after its delivery from DNA and MVA vectors in a mouse model, employing Nef as a tool to evaluate the impact of subtype B vs BF variability in vaccine strategies. We found that NefBF delivered from DNA and MVA vectors generated a response of high specificity with low cross-reactivity against subtype B [42]. Taking in account the high HIV variability, the aim of the present study was to analyze if the co-administration of genetic adjuvants such as IL-12 and/or GM-CSF, from DNA vectors, during the priming immunizations in DNA/MVA schemes was able to modulate not only the magnitude, but also the breadth and quality of the NefBF-specific immune response generated, as well as the cross-reactivity against other Nef subtypes.

## Results

### 1. IL-12 and GM-CSF during the priming phase enhanced the cellular immune response against NefBF

In a previous study, we evaluated the Nef-specific cellular immune response generated after immunizing mice with DNA and MVA vectors expressing Nef from the circulating recombinant form CRF12_BF (NefBF) (three DNA doses as prime followed by one MVA boost). Using this strategy, highly specific responses were generated with low cross-reactivity against Nef from B subtype (NefB) [42].

Due to the necessity and the relevance of generating cellular immune responses with higher amplitude in the context of HIV-1 diversity, we decided to explore strategies able to enhance the response generated after NefBF immunization and to investigate the possibility of increasing cross-reactivity against other subtypes. We hypothesized that the inclusion of cytokines such as IL-12 and GM-CSF, which play a key role in the generation of a Th_1_-type immune response [35], [37], [38] and were previously used to enhance immune responses generated from DNA vectors [31], [40], [41], would benefit the specific T cell responses generated in terms of not only the magnitude, but also breadth and quality.

**Figure 1:**
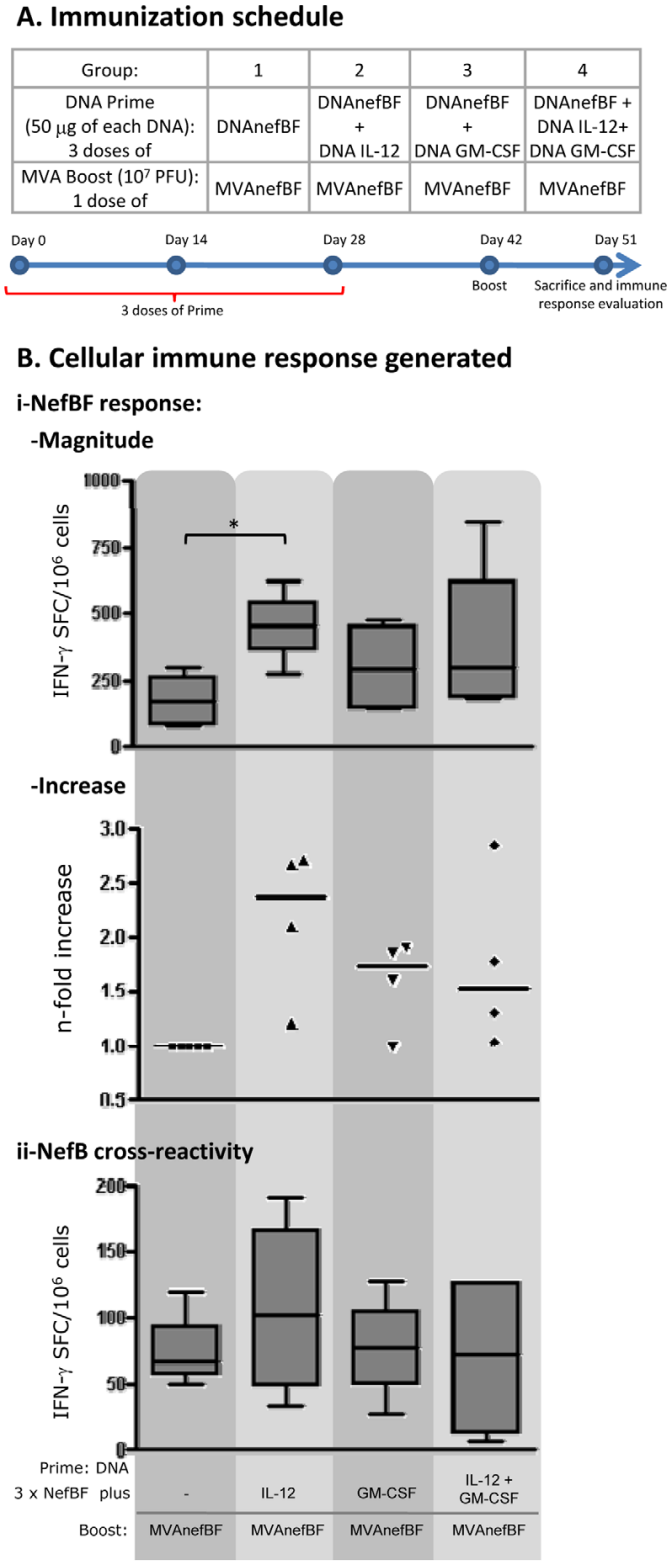
IL-12 and GM-CSF incremented the magnitude of the specific immune response. **A**) Immunization schedule: Groups of 4 Balb/c mice received the different immunization regimes depicted. Immunization doses (both priming and boost doses) were spaced by 14 days. **B**) Nine days after the MVAnefBF boost dose, cellular immune responses against a pool of peptides representing **i-** NefBF or **ii-** NefB were evaluated in the spleen, by quantifying specific IFN-γ secreting cells by ELISPOT; **i-** Magnitude of the NefBF (homologous) response; n-fold increase represents the increments in the responses generated in relation to those found in the control group. **ii-** Magnitude of the cross-reactivity responses detected against NefB. Data represent the median values found in 4 to 5 independent experiments. Background values found in negative control wells were subtracted (5 to 50 spots/10^6^ cells). Mann Whitney test was applied, comparing NefBF responses between groups (* p<0.05). For the box and whisker plots, the horizontal line represents the median, the boxes represent the interquartile range and the whiskers represent the minimum and maximum values.

Immunization schemes consisted of three DNA priming doses, spaced each other by 14 days, containing a NefBF expressing DNA (DNAnefBF, 50 µg) plus IL-12 or/and GM-CSF expressing plasmids (IL-12; GM-CSF, 50 µg of each one), separately or in combination. Mice primed only with DNAnefBF (without cytokine expressing DNAs) were used as the control group. Fourteen days after the last DNA priming dose all the groups received a booster dose of MVAnefBF (Figure 1A).

Nine days after boosting, mice were sacrificed and the immune response was evaluated in the spleen by quantifying the IFN-γ secreting cells by ELISPOT. In figure 1Bi (upper panel) the magnitude of the immune response found in the different groups against a pool of peptides representing the whole NefBF protein is shown. The data shown correspond to median values obtained from four to five independent experiments. The highest magnitude of NefBF-specific immune responses was found in those groups immunized with IL-12 alone in which a statistically significant increment in magnitude was detected, when compared to the control group (without adjuvants) (Mann Whitney test p = 0.031) (Figure 1Bi). IFN-γ SFC/10^6^ cells median for IL-12 group was 457 [range 275–622] versus 169 [range 79–296] in the control group. In the groups receiving GM-CSF alone or in combination with IL-12, we also detected an improvement in the response generated, where IFN-γ SFC/10^6^ cells median for GM-CSF and GM-CSF+IL-12 groups were 289 [range 142–477] and 296 [range 176–845], respectively. However, a higher variability among the different independent experiments was found in these groups, thus no statistically significant differences could be detected (Fig. 1Bi).

Then, we calculated the n-fold increments in the magnitude of the NefBF responses found in the cytokine-DNA groups as: the responses found in the cytokine-DNA group/response detected in the control group (Figure 1Bi). The highest increment was found in the IL-12 groups (median 2.38 [range 2.71-1.21]), whereas 1.73- [range 1–1.91] and 1.53-fold [range 2.86-1.05] increments were registered for the GM-CSF and GM-CSF+IL-12 groups, respectively. Additionally, the NefBF-specific IL-2-secreting cells were also evaluated in these groups by ELISPOT. We found an enhanced response in the three groups with adjuvants, but the highest response was found in IL-12+GM-CSF group (Figure S1, Student's t-test, p = 0.003). When the cross-reactivity against NefB was evaluated, responses 2- to 4-fold lower than the homologous response were detected (Figure 1Bii). The highest responses directed towards the heterologous NefB peptides were also found in IL-12 group (median 103 [range 34–191]) but without reaching statistical significance when compared to the control group (median 67 [range 49–120]) (Figure 1Bii).

Taking together, these results show that IL-12 and GM-CSF resulted effective genetic adjuvants to enhance the specific immune response generated against NefBF. However, out of the different immunization schemes analyzed, the administration of DNA IL-12 alone induced significantly higher magnitude increments against the homologous NefBF response.

### 2. Groups which received IL-12 or GM-CSF induced cellular immune responses with higher breadth

The results depicted in the previous section showed the capacity of IL-12 and GM-CSF to increment the magnitude of the homologous NefBF response. Undoubtedly, an immune response directed against HIV with higher breadth, having the ability to recognize multiple antigens/epitopes and/or different variants of the peptides targeted, is a desired feature to be hold by a vaccine formulation. Therefore, we considered important to do a deeper evaluation of the ability of these adjuvants to improve the breadth of the generated response by analyzing in first place which regions of the NefBF protein were targeted, and then the level of cross-reactivity against different HIV subtypes.

**Figure 2:**
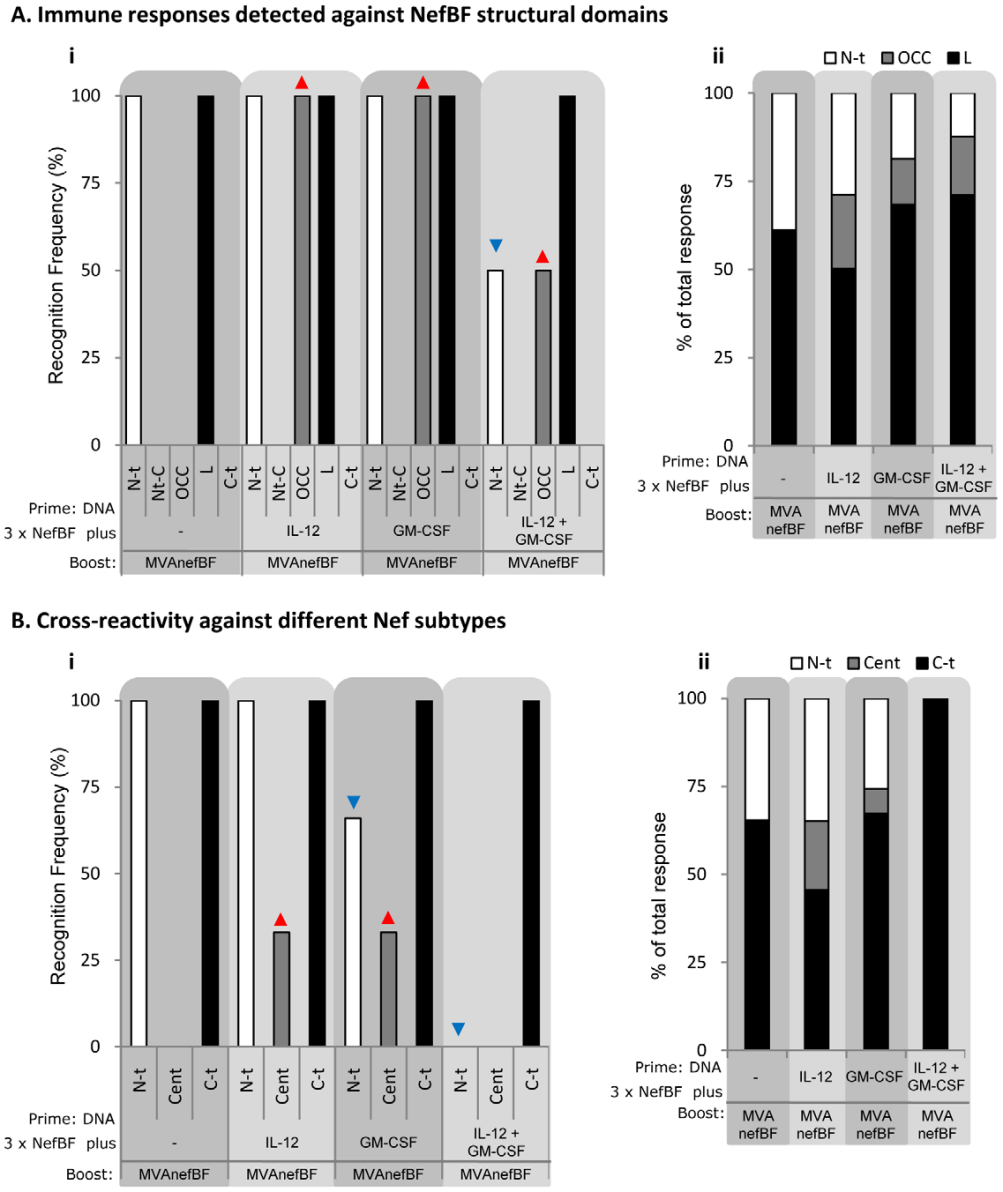
The use of DNA-IL-12 or DNA-GM-CSF during the priming phase in DNA/MVA schemes enhanced the breadth of the cellular response induced. **i** Recognition frequency represent the proportion of experiments in which a positive IFN-γ ELISPOT response was found for each protein domain. **ii** Bars show the percentage of the whole response (obtained from the sum of the responses directed against each of the five peptide pools) which is directed towards the different Nef domains. Red and blue arrows denote increments and decreases compared to control group respectively. **A**) NefBF peptides were mixed in five pools representing the structural domains of Nef protein: N-terminal domain (N-t, 1–83 aa), N-terminal Core (Nt-C, 84–115), Oligomerization domain+Central Core (OCC, 116–159), the Loop of the protein (L, 160–187) and C-terminal (C-t, 188–205). Data are from two or three independent experiments for DNAck or control groups, respectively. **B**) PTE peptides were mixed in three pools, representing N-terminal (N-t, 1–83 aa), Central (C, 84–159) and C-terminal (C-t, 160–205) Nef domains. Data are from three or four independent experiments for DNAck or control groups, respectively.

We divided the NefBF peptide set in pools representing the different protein domains and these pools were used as stimulus in the IFN-γ ELISPOT assay. The pools represented the N-terminal domain (N-t, comprising from the first amino acid (aa) to 83), the N-terminal Core (Nt-C, 84 to 115), the Oligomerization domain and Central Core (OCC, 116–159), the Loop of the protein (L, 160–187) and the C-terminal domain (C-t, 188–205) [43]. First, the recognition frequency of the different protein domains was analyzed (Figure 2A). Interestingly, it was found that the three groups receiving any of the genetic adjuvants recognized a higher number of protein domains. Mice from the control group (receiving NefBF alone during the priming) recognized the N-t and the L domain in all experiments. The responses with higher amplitude were detected in the groups which received IL-12 or GM-CSF as cells from these mice recognized the N-t and L domains plus the pool representing the OCC domain (Figure 2Ai). However, when both cytokines were co-administered, positive responses against the N-t and OCC pools were detected with a minor frequency. In order to further illustrate differences in breadth of the response induced among groups, the proportion of the total response directed against each of the targeted peptide-pools was analyzed (Figure 2Aii). The highest breadth was detected in the IL-12 group in which the magnitude of the response was distributed more evenly along the three protein-regions targeted, whereas in the other two groups (GM-CSF and IL-12+GM-CSF) the response was more focused against the L domain, on detriment of the other recognized domains.

Then, having observed that the cytokines incremented the amplitude of the specific response induced against the antigen, we proceeded with the analysis of the cross-reactivity against other subtypes. For this, we performed an ELISPOT assays using the Nef PTE (Potential T-cell Epitopes) peptide set, obtained from NIH AIDS Reagent Program, in which a total of 127 peptides representing A, B, C and non-ABC HIV-1 subtypes are included. In this case, the peptide set was divided in three pools, N-terminal pool (first aa to 83, N-t), Central pool (aa 84 to 159, Cent) and C-terminal pool (aa160 to 205, C-t). As it was found for the homologous peptides, the administration of IL-12 or GM-CSF generated a cellular response that cross-recognized a higher number of peptide-pools (Figure 2Bi). However, when both genetic adjuvants (IL-12 and GM-CSF) were co-inoculated, this improvement was lost. When the percentage of the response directed against each pool was analyzed (Figure 2Bii), again the IL-12 group showed the response with the higher breadth.

Data from this section demonstrate that the utilization of the cytokines separately improved the breadth of the response induced, obtaining better results with IL-12. Importantly, these results were evidenced by evaluating both the recognition frequency of homologous and heterologous peptides as well as the percentage of the total magnitude directed towards the different protein regions targeted.

### 3. Mapping of the NefBF peptides targeted after immunization and the cross-reactive NefB counterparts

Our next aim consisted in the identification of the individual Nef peptides targeted. We employed a matrix-peptide strategy where each peptide was represented in two different peptide pools, allowing for the identification of the reactive peptide by the detection of positive responses in the two pools [20]. Two matrixes were constructed: one with Nef BF peptides (BF matrix; consisting of 12 pools of 6 peptides each one) and the other one with NefB peptides (B matrix; consisting of 14 pools of 7 B peptides each one). These matrix-peptide pools were used as stimulus in IFN-γ ELISPOT assays and in splenocyte cultures to later evaluate the specific IFN-γ secreted in the supernatants after 72 h by ELISA. Seven putative positive peptides using the BF matrix were detected: BF2, BF3, BF9, BF26, BF27, BF32 and BF33; and three using the B matrix: B41, B49 and B84 (*data not shown*). Afterwards, ELISPOT assays using individual peptides were performed to corroborate the putative targeted peptides, and only six BF and two B peptides were confirmed: BF2, BF3, BF26, BF27, BF32, BF33, B41 and B84. Positive responses for overlapped consecutive peptides, as observed for BF2–BF3, BF26–BF27 and BF32–BF33 peptides, were assumed to be representative of the same epitope thus the peptides with the higher response were selected to perform the next experiments: BF3, BF26 and BF33.

**Figure 3:**
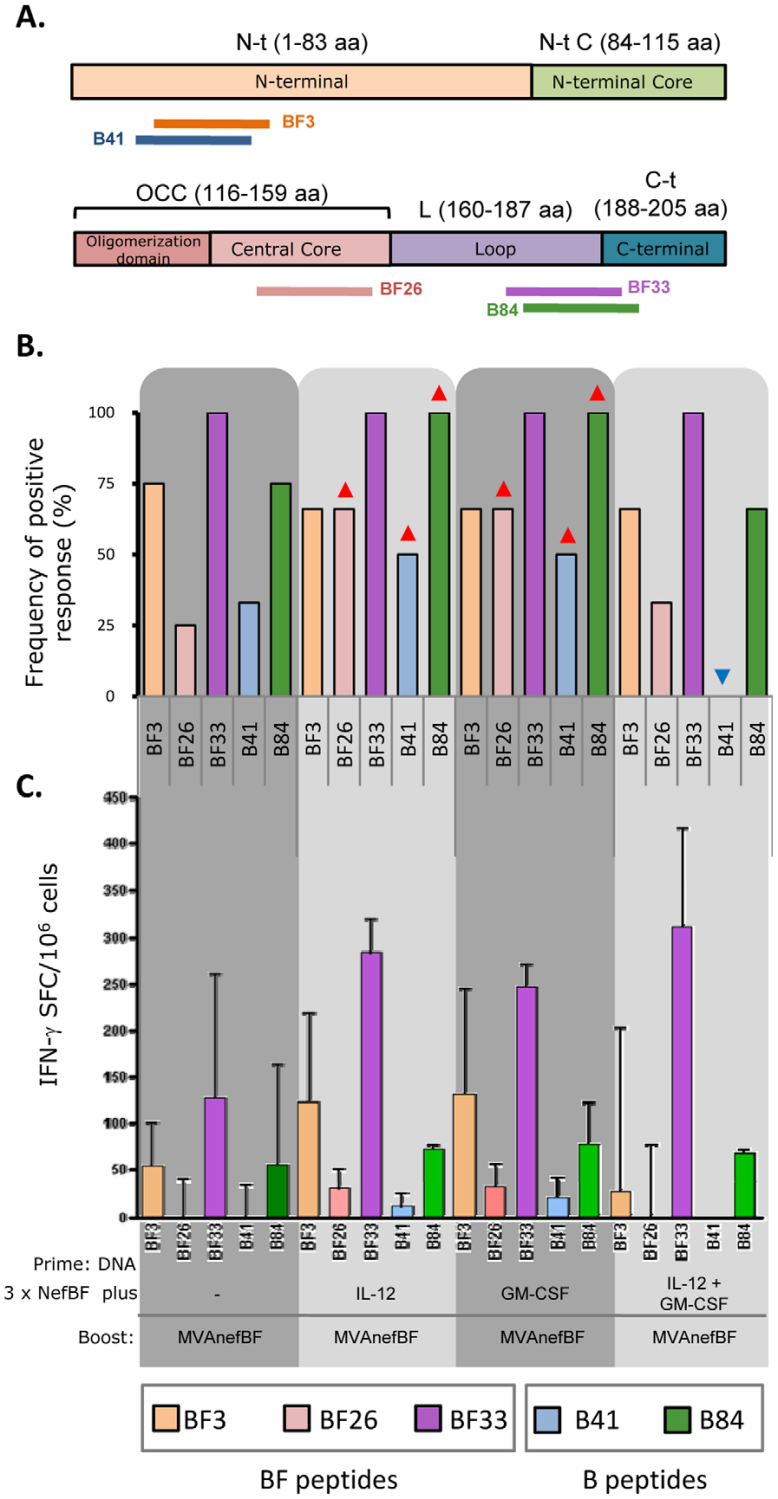
NefBF and NefB peptides targeted. **A**) Nef Structural domains are represented and the localization of the targeted BF and B peptides is indicated. **B**) Recognition frequency represents the proportion of experiments in which a positive response was found for each indicated peptide. Red and blue arrows indicate differences (increments and reductions, respectively) compared to the control group. **C**) Bars show the magnitude median of the responses (number of IFN-γ secreting cells, evaluated by ELISPOT) detected in each group towards the different identified peptides. Data are from two to four independent experiments for the different groups and peptides.

Figure 3A depicts the localization of the individual BF and B peptides targeted within the different Nef protein domains, thus identifying the immunogenic peptides responsible for the responses showed in Figure 2. Figures 3B and 3C show the analysis of the recognition frequency of each individual peptide in the whole set of experiments and the percentage of the response (out of the total magnitude) directed towards these peptides. We found that the BF33 peptide, located on the loop of the protein (aa 179–193), resulted highly immunodominant, being the most frequently recognized (Figure 3B). Also, this peptide was the most immunogenic, inducing the highest magnitude in all groups (Figure 3C).

All groups also recognized the BF3 peptide, located on the N-terminal domain (aa 11–26), but with lower frequency and magnitude than the BF33 peptide, evidencing a lower immunogenicity for this region of the NefBF protein (Figure 3B and C). The BF26 peptide, located on the central core (aa 140–155), was also recognized by the four groups, but with the lowest frequency and magnitude (Figure 3B and C), suggesting to have subdominant feature in this mice model. Interestingly, IL-12 or GM-CSF administration induced a higher frequency of recognition for the BF26 subdominant peptide (Figure 3B). Both frequency and magnitude of recognition of the BF peptides were similar for groups in which IL-12 or GM-CSF was delivered. However, when both genetic adjuvants were simultaneously applied a significant reduction in the amplitude was observed as BF33 accounted more than the 80% of the magnitude of BF the response (Figure 3C).

Regarding the cross-reactivity recognition pattern, the B84 peptide (aa 181–195), homologous to BF33, was recognized by all the groups, while the B41 peptide (aa 9–23), homologous to BF3, was not recognized by animals from the IL-12+GM-CSF group. In line with the results observed for the homologous peptides, IL-12 and GM-CSF (administered alone, not in combination) improved the frequency of recognition of the heterologous B peptides (Figure 3B).

After the analysis of the Nef immunogenicity pattern, it was demonstrated that by delivering IL-12 or GM-CSF as genetic adjuvants during the priming doses, it is possible to induce cellular responses with an improved breadth in terms of higher frequency of positive responses as well as a more balanced response where the magnitude was more evenly distributed towards the different targeted epitopes.

### 4. Peptide sequence analysis

**Figure 4:**
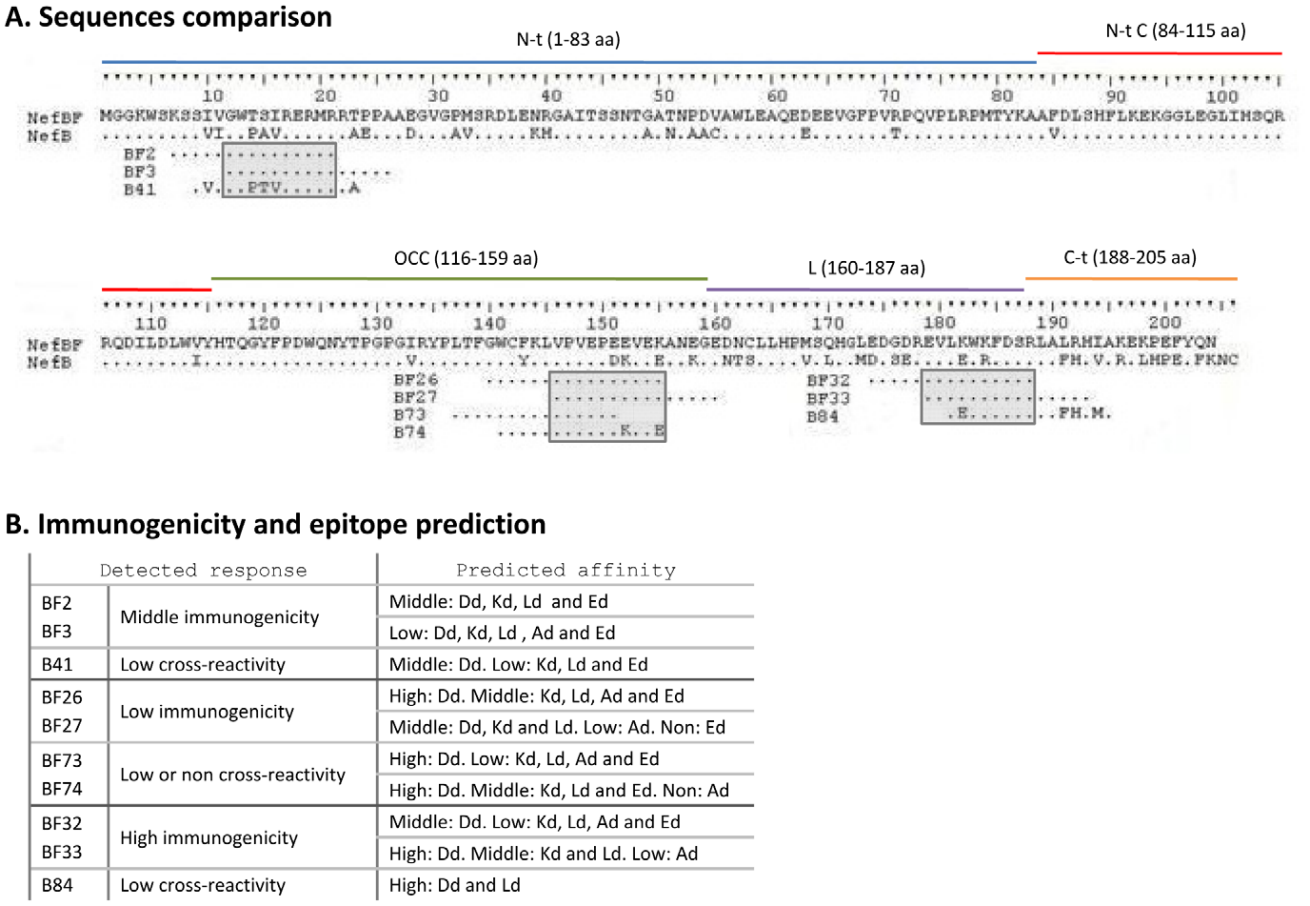
Sequence analysis and localization of B and BF peptides targeted. **A**) Comparison between amino acid sequences of NefBF and NefB proteins, and BF and B peptides targeted. Grey boxes indicate the overlapping peptide sequences between the BF recognized peptides, suggesting the potential epitope. **B**) Peptide immunogenicity of the response found is compared with the predicted MHC affinity. Prediction analysis were made with the SYFPEITHI Epitope Prediction (Class I: Ld and Kd; Class II: Ad and Ed) and PREDEP (Class I: Dd) prediction softwares.

After the identification of the individual targeted peptides, we proceeded with the analysis and comparison of their sequences. In figure 4A, it is described the complete aa NefBF sequence in comparison with the NefB one. Sequences and localization of the BF and B peptides and of the consecutive peptides that gave a positive response are shown. The peptide responsible for the positive responses found against the N-t peptide pool was BF3 which differ from its heterologous counterpart B41 in five aa. Two of them were located at each end of the peptide, while the other three were almost in the middle of the overlapping sequence between BF2 and BF3, where the potential minimal epitope is probably located, marked with a gray box in the figure 4. Thus, these three aa changes located near the middle of the epitope might probably reduce the recognition of B41 by the T-cells. In the case of the peptides located within the central core of the protein (BF26–BF27 and the heterologous counterparts B73–B74), we found that peptide B73 does not include the whole overlapping sequence between BF26 and BF27 (potential minimal epitope) what might explain its abrogated recognition. In the case of B74, the peptide covers the overlapping sequence but has two aa changes: one located on the carboxi-terminal end and the other near the middle of the peptide, what might explain the absence of cross-recognition.

When we analyzed the BF33 peptide sequence and compared it to the heterologous peptide B84, four aa changes were detected in the B sequence. Three of them were located in the carboxi-end of the peptide and the other change (K to E) was located in a more central position of the potential epitope. None of these changes seemed to avoid recognition given the cross-reactivity observed in all experiments. However, it must be noted that the magnitude of B84 responses were always significantly lower compared to the homologous (BF33) response.

Then, we performed an analysis of epitope prediction based on the probability of binding to the H-2d MHC class I and II alleles of the peptides used in our T-cell assays by employing software tools [44] (Figure 4B). The analysis for the immunodominant BF33 peptide indicated a high probability of being presented by H-2 Dd MHC class I, middle scores to bind H-2Kd and Ld, and low score to bind the H-2Ad class II molecule.

On the other hand, the subdominant BF3 showed low affinity binding scores to any MHC allele. Conversely, high score for the Dd allele and middle scores for Kd, Ld, Ad and Ed alleles were obtained for the BF26 peptide. However, we were unable to detect a high response against this peptide.

Finally, the B84 binding prediction to MHC alleles gave a good score to bind to MHC class I alleles. In spite of this, responses against this peptide were low. Probably due the aa differences with the BF33 counterpart peptide, present in the immunogen- constructs.

A prediction of the potential epitopes present on the whole NefBF sequence expressed from the DNA and MVA vectors was also performed. The analysis showed high affinity scores to the H-2 Dd and Ed alleles (class I and II respectively), for sequences comprised from aa 181 to 195, the region in which the BF33 peptide (aa 179–193) is located. The analysis also indicated that a sequence comprised between aa 7 to 25 have a high score of binding to Kd (class I) and middle score of binding to Ed (class II), in concordance with the middle immunogenicity that was found for the BF3 peptide (aa 12–26). These predictive values coincide with our results. However for other sequences that also showed high affinity to the other H-2d alleles (68–77, 135–144 to class I; 31–45 to class II) positive T-cell responses could not be found.

Therefore, these analyses indicate that the recognition pattern of the immunogenic peptides identified could be explained by the coincidence or divergence of the aa composition of the peptides. However, the comparison between the immunogenic predictions either for the individual peptide sequences or for the complete sequence expressed from the vaccine vectors, overlap only in part with the results obtained from *in vivo* experiments. Although reactivity of murine T cells does not directly translate to humans, the underlying principles of the molecular interactions involved in triggering T cell responses are the same in both species, thus it must be expected that results obtained here would be potentially extrapolated to a human scenario.

### 5. IL-12 and GM-CSF improved the quality of the cellular response against NefBF

There are several studies exploring correlation between different characteristics of the specific T-cell response and the control of aids progression. Certain T-cell properties, such as their polyfunctionality or their capacity to proliferate as well as the T-cell avidity, have been directly correlated with enhanced anti-viral immunity. In this sense, it has been described in different models (such as SIV, cohorts of chronically HIV-infected patients, controller patients and HIV-exposed seronegative persons) that the polyfunctional CD8^+^ T-cell responses, in terms of capacity to secrete multiple cytokines, may play an important role in the control of viral replication, with a direct correlation with protection [45], [46].

**Figure 5:**
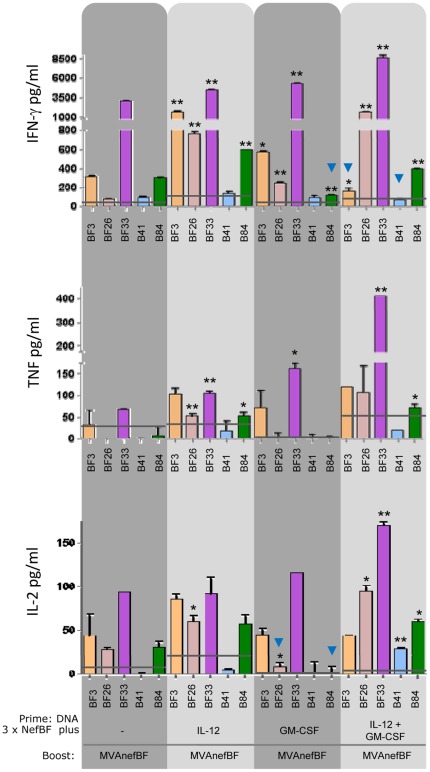
IFN-γ, TNF and IL-2 secretion pattern after re-stimulation with homologous or heterologous peptides. Spleen cells from mice immunized with 3xDNA/MVAnefBF plus IL-12 or/and GM-CSF (as depicted in Figure 1) were re-stimulated during 72 h with the indicated peptides. Levels of IFN-γ, TNF and IL-2 secreted in supernatants were quantified by ELISA. Bars represent the average levels of cytokines secreted in cell supernatants from pooled samples of triplicate cultures. Negative controls (RPMIc) values were subtracted (<130 pg/ml for IFN-γ, <30 pg/ml for TNF and <14 pg/ml for IL-2). Cut-off levels (RPMI values+2xSD) are shown by horizontal grey lines. Significant differences between responses found in control vs the other three groups are indicated with ** (p<0.01) and * (p<0.05).

Thus, in order to address a deeper study of the immune response generated with these immunization schemes, we studied the cytokine secretion pattern induced against the different Nef peptides targeted. To this aim, the secretion of IFN-γ, TNF and IL-2 in the supernatant of cell-cultures re-stimulated for 72 h with the targeted peptides was evaluated by ELISA (Figure 5). The Pattern of cytokines secreted against the immunodominant BF33 peptide showed that statistically significant increments -compared to the control group- in the levels of IFN-γ and TNF were detected in the three groups which received any of the adjuvants. IL-2 levels were also incremented in the three groups, but significant differences were only detected in the IL-12+GM-CSF group. The improvement in the T-cell responses generated in relation to the multiple cytokines secreted was more evident in the analysis of the response against the subdominant BF26 peptide. Whereas in the control group only a positive IL-2 response, slightly above the cut-off value was detected, significant positive levels of the three cytokines were found in both the IL-12 and IL-12+GM-CSF groups. In relation to the cross-reactivity response detected, the pattern of cytokines secreted against the B84 peptide was significantly improved in both the IL-12 and IL-12+GM-CSF groups in comparison to the control, detecting significant increments in the levels of the three cytokines (see Figure 5).

Another quality of the T-cell responses that correlated with effectiveness in their effector functions is the cellular avidity for the antigen. In this line, a recent study demonstrated that HIV controllers harbor a pool of memory CD4^+^ T cells with the intrinsic ability to recognize minimal amounts of Gag antigen, which may explain how they maintain an active antiviral response in the face of very low viremia [47].

**Figure 6:**
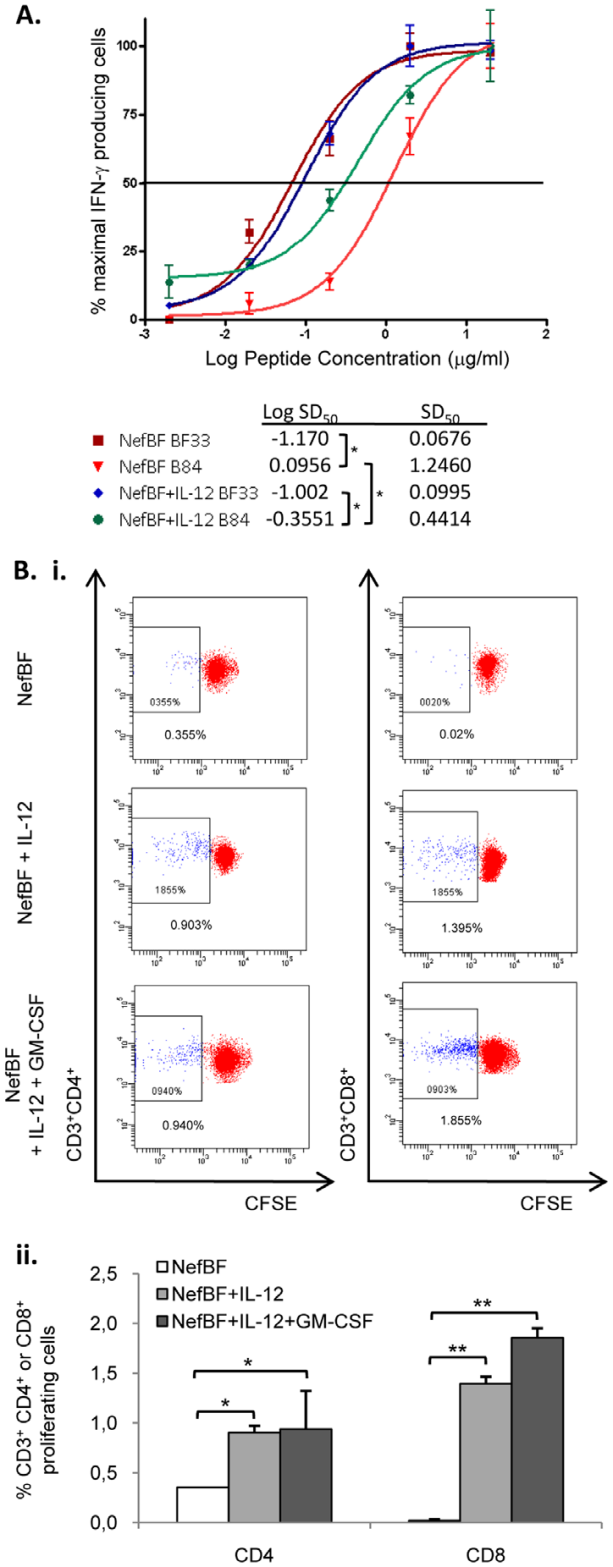
IL-12 and GM-CSF improved the quality of the cellular immune response. **A**) Functional avidity of the BF33- and B84-specific T-cells: Groups of 4 mice received the immunization schemes depicted in Figure 1 for the group 1 (NefBF alone) and group 2 (plus IL-12 during the priming phase). Nine days after the MVA boost, T- cell avidity against BF33 and B84 peptides was evaluated using serial dilutions of the peptides. Peptide concentrations used ranged from 20 to 0.002 µg/ml. SD_50_: sensitizing dose of peptides required to yield 50% maximal T-cell triggering of IFN-γ production. **B**) Spleen cells from the indicated group were evaluated for their proliferative capacity against the BF33 peptide, and the identification of the specific T-cell subset was performed. For this, cells were dyed with CFSE and stimulated with BF33 peptide during 5 days and then staining with surface antibodies was performed (CD3, CD4 and CD8). **i-** Representative dot plots. Numbers inside the graphs represent the average values for duplicated samples. **ii-** Bars represent the average of duplicated samples plus SD. Background values obtained in cell-cultures stimulated with RPMIc, were subtracted in each case. T test was applied, *p<0.05, * * p<0.005.

Thus, our next aim was to explore the functional avidity of the T-cells in our model. In these experiments, the responses found in the control vs the IL-12 group were compared, analyzing the avidity of the T-cells against the BF33 (same sequence as the immunogen vectors) or the B84 peptide (with 3 aa changes, see Figure 4), in order to define if the recognition pattern differed between homologous or heterologous responses. T-cell avidity was analyzed performing ELISPOT assays at different peptide concentrations, making serial peptide dilutions (Figure 6A).

Intra-group analysis indicated that the sensitizing dose of peptide concentration required to yield 50% of the maximal T-cell IFN-γ response (SD_50_) was significantly higher, indicating a lower avidity, for the B84 compared to the BF33 in both the control group (SD_50_: BF33 [0.0676 µg/ml] vs B84 [1.246 µg/ml]; p<0.0001) and in the IL-12 group (SD_50_: BF33 [0.0995 µg/ml] vs B84 [0.4414 µg/ml]; p<0.0001). These results demonstrated that a higher T-cell avidity for the homologous BF33 peptide compared to the heterologous B84 peptide was found in both groups analyzed.

An intergroup analysis revealed statistically significant differences in the T-cell avidity against the B84 peptide. Interestingly, the highest avidity against this peptide was found in the group receiving IL-12 as adjuvant. In this group the value of the SD_50_ was 3-fold lower compared to the control group (control group: B84 = 1.246 µg/ml vs IL-12 group: B84 = 0.4414 µg/ml, p = 0.001). These results indicate that the administration of IL-12 improved the cross-reactive response generating a T-cell recognition of the heterologous B84 peptide with higher avidity being able to recognize concentrations three-fold lower of the B peptide ligand in comparison to the T cells from the control group.

Mean spot sizes, as a way to measure the IFN-γ secretion potency of the specific T-cells against BF33 or B84 (using the ImmunoSpot software) were also analyzed. In correlation with the higher avidity for the homologous BF33 peptide, the spot size was higher when cells were re-stimulated with the homologous peptide: B84 median spot size was 2.65-fold lower than BF33 spots [range 6.3-1.5], without differences between both immunized groups.

Finally, the proliferative capacity of the BF33 T-cell responses induced after immunization was analyzed. In these assays, cryopreserved splenocytes from control group, Il-12 and IL-12+GM-CSF groups were used. It must be noted that in previous experiments performed in our lab, a good correlation between T cell assays performed with fresh and cryopreserved cells was always registered [42].

Proliferation was evaluated on thawed and then rested cells by the CFSE dilution assay. Briefly, after five days of stimulation in the presence of the peptide or medium alone, viable CD4^+^ and CD8^+^ T-cells that had proliferated were determined by flow cytometry. As the epitope prediction analysis anticipated (Figure 4), CD4^+^ and CD8^+^ proliferating BF33-specific cells were found (Figure 6Bi and ii). Significant increments in the proportion of proliferating CD4^+^ and CD8^+^ T-cells were observed in IL-12 and IL-12+GM-CSF groups, compared to the control group (Figure 6Bii). Only 0.355% of CD4^+^ T-cells from the control group proliferated, whereas values found for IL-12 and IL-12+GM-CSF groups were 0.903 and 0.940% respectively, rendering a 2-fold increment in the CD4^+^ proliferating cells (Figure 6Bi, left panel). When the CD8^+^ T-cells were analyzed, no proliferation was found in the groups without adjuvant. However, 1.395% and 1.855% of CD8^+^ T-cells proliferated in IL-12 and IL-12+GM-CSF groups, respectively (Figure 6Bi, right panel).

Altogether, data from these experiments showed that by delivering of IL-12 or IL-12+GM-CSF from DNA vectors in DNAprime-MVAboost regimes, the quality of the T-cell response induced could be improved. This fact was confirmed by the findings showing that a more complex array of cytokines was produced against different peptides and both CD4^+^ and CD8^+^ specific T-cells showed superior proliferative capacities. Moreover, we could demonstrate that, after IL-12 administration, T-cell avidity against the heterologous B84 peptide was significantly improved.

## Discussion

More than a dozen antiretroviral drugs have been developed since HIV/aids was first described, leading to a significant decrease in mortality associated with aids over the years and increasing the life quality of HIV infected people. These improvements have occurred especially in developed countries, where the therapy is available for all HIV^+^ individuals, whereas only during the last years ARV therapy have begun to be implemented in developing countries [48]. Despite these advances, HIV continues to spread around the world with an estimated rate of 7400 new infections per day [1]. A critical obstacle in HIV vaccine development is how to induce immune responses able to overcome the intra- and inter-subtype viral variability. In Argentina, the epidemic is characterized by the co-circulation of subtype B variants and BF recombinant forms, mostly related to the CRF12_BF [4], [5]. One way to conquer the variability challenge could be the design of multivalent vaccines, based on formulations including antigens from the different HIV subtypes that circulate in certain geographic areas. In fact, the RV144 clinical trial (performed in Thailand) included both antigens from subtype B (widely distributed around the world, mainly in developed countries) and CRF01_AE, prevalent in Thailand [25]. Different preclinical studies also described the use of different vectors expressing proteins from different clades [15], [29], [49].

During the last years, our laboratory have studied different biological aspects (associated with the replication and immunogenicity) of the CRF12_BF and its related variants, with particular emphasis in viral regions which differed from the parental B subtype [20], [42], [44], [50], [51]. In particular, we have recently reported the construction of viral and DNA vectors expressing NefBF and EnvBF. These vectors were administered by different immunization schedules, in a mice model, inducing a specific BF response with low B cross-reactivity [42], [44]. It is not completely defined which characteristics of the T-cell immune response are required to fight against the HIV infection, but several studies point to a high magnitude, breadth and polyfunctionality as desirable features for a vaccine candidate [23], [24]. To achieve these T cell characteristics we considered the utilization of adjuvants to generate an enhanced and improved response.

DNA plasmids expressing murine IL-12 and GM-CSF as potential adjuvants to enhance the immune response generated by DNA-prime/MVA-boost immunization schemes were used. When the cytokine modulation effects on the magnitude of the response against the total NefBF protein was analyzed, the higher increments were found when IL-12 was administrated alone (median of 2.38 fold vs the control group). On the other hand, when GM- CSF was administered alone or combined with IL-12, the observed increments were lower (medians of 1.535 and 1.73 fold increments compared to control). Surprisingly, in the groups which received both genetic adjuvants we could not find a synergism nor even an additive effect of the cytokines in relation to the final magnitude detected.

Different previous reports accounted for the benefits of IL-12 administration in the context of HIV antigens [30], [41]. For instance, in a recent macaque study, the administration of DNA expressing SIV antigens plus DNA expressing IL-12, boosted with Ad5, generated a reduction of the median SIV peak and set point viral loads [52]. However, IL-12 modulating effects on the amplitude of the response generated after vaccination and its impact on cross-reactivity frequency against different HIV subtypes have not been studied previously. When we analyzed the amplitude of the response generated, by evaluating the frequency of recognition of the different NefBF protein domains, differences among groups were detected. Interestingly, all the groups gave a positive response against the N-t and L domains, but the OCC domain was only recognized in those groups which received any of the adjuvants, indicating that the highest breadth was obtained in the groups which received any of the adjuvant-treatments. To denote, mouse groups in which IL-12 or GM-CSF were applied alone recognized the N-terminal, OCC and L domains with the highest frequency, and the percentage of the magnitude against these responses was more evenly distributed (Figure 2A). Cross-reactivity against Nef of different subtypes was evaluated with the use of PTE peptides (representing Nef sequences of A, B, C and non-A,B,C subtypes). In this case, the best results in terms of recognition frequency and magnitude percentage distribution against the different peptide-pools, were obtained in the IL-12 group.

Regarding the consequences that the use of different adjuvants could have on the breadth of the T-cell responses induced, it has been previously reported that IL-12, also administered from a DNA vaccine in conjunction with DNA expressing SIV Gag, increased the specific cellular response induced [53]. In concordance with our results, the authors demonstrated a significant correlation between the magnitude and breadth of the T-cell response induced in the vaccines. Another recent study in which soluble CD40 ligand was used as an adjuvant, also indicated an improvement in both the magnitude and breadth of the T-cell response generated by the use of the adjuvant in conjunction with DNA expressing HIV antigens during the priming doses in DNA/MVA or NYVAC immunizations [54].

When the response was split up into the single peptides targeted, it was found that the majority of the response was directed towards the BF33 peptide (aa position 179–193), located in the intersection of the loop and the C-terminal domains of the protein. This peptide was recognized both by CD4^+^ and CD8^+^ T-cells, as the prediction analysis anticipated and as Santoro Rosa *et al.* described previously [55]. Also, this peptide sequence had been previously reported to be immunogenic in Balb/c mice in different reports [13], [55], [56], [57]. The cross-reactivity (against B84 peptide) was improved by the employment of the adjuvants, but this response always remained lower than the homologous response. The aa changes in this protein portion, located at both ends of the peptide, did not ablate but depressed the recognition by the T-cells. These changes probably generated weaker interaction between the peptide and MHC, thus failing in the activation of T lymphocytes.

The fine mapping of the rest of the NefBF T-cell epitopes targeted, included subdominant peptides as the BF3 (aa position 12–26 aa) with some cross-reactivity with B41 (9–23 aa). These peptides are located in the N-terminal portion of the protein. There are five aa changes between them, three located in the central part of the sequence, which probably prevented the peptide recognition by the TCR or its interaction with MHC molecules. To denote, this Nef region has not been described previously to be immunogenic in Balb/c mice but for one earlier study where, in coincidence with our results, the authors described a low immunogenicity for it (<20% of responder mice) [13]. The other BF peptide mapped was BF26 (aa position: 140–155), for which we registered the lowest responses. Again, the only previous report in which this region was cited to be immunogenic in Balb/c mice was the study of Hinkula et.al., reporting for it a low proportion of responsiveness (<20%) [13].

When we evaluated the IFN-γ, TNF and IL-2 secretion pattern for, data showed that, by co-delivering IL-12 or IL-12+GM-CSF from DNA vectors in DNAprime-MVAboost regimes, the quality of the T-cell response induced could be improved. As a result, a more complex array of cytokines was produced against the different peptides targeted and, importantly, both CD4^+^ and CD8^+^ specific T-cells from these mice groups showed superior proliferative capacities.

When T-cell functional avidity was analyzed, no differences between IL-12 and control groups were found against the immunodominant BF33 peptide. But interestingly, we demonstrated that in the IL-12 group, a significant higher avidity was found against the heterologous B84 peptide. Thus, we could determine that mice from this group not only had an increment in the frequency of positive responses against the B peptides (Figure 3B) but also exhibited an enhanced T-cell avidity against at least one (B84) of the cross-reactive B peptides. As mentioned above, comparing the BF33 vs B84 peptide sequences four aa substitutions were located at the ends of the B84 peptide allowing the cross-recognition, but decreasing both the magnitude and avidity of the cross-reactive T-cells. In a previous work from our lab, no differences were found in T-cell avidity comparing EnvB and EnvBF peptides (13BF vs 16B, located on the C1 region, aa 61 to 75) that differed in only two aa located at one end of the peptides. In line with this result, no differences in the magnitude of the responses against those peptides were found [44]. Limited cross-reactivity against altered peptide ligands, with an aa substitution in a central epitope position, was previously demonstrated in a lymphocytic choriomeningitis virus (LCMV) model, in which the authors also showed that these type of aa changes were only able to mediate moderate anti-viral protection [58].

The highest avidity against the B84 peptide (representing a cross-subtype response) found after IL-12 co-administration may be explained by IL-12 properties described in earlier studies where it is shown that this cytokine promotes an enhanced synapse formation between APCs and T-cells, leading to the recognition of weak peptides against which the T cells would be normally unresponsive [59]. Moreover, another study also described that the generation of high-avidity effector CTLs is IL-12 dependent [60]. Regarding the use of other adjuvants and their effects on T-cell avidity, it has been recently published that the use of three TLR ligands (MALP2 (TLR2 ligand), poly(I:C) (TLR3) and CpG (TLR9)) as adjuvants, augmented the quality of the Env specific T-cell response amplifying their functional avidity [61].

In conclusion, this study shows that by including cytokines such as IL-12 and/or GM-CSF expressed from DNA vectors during the priming doses of DNA/MVA schemes, the cellular response generated against the delivered antigen (NefBF) could be improved. A summary of the results obtained along this study is presented in Table 1. The use of IL-12 showed the best results both generating significant increases in the magnitude of the induced cellular immune response and also superior T-cell properties in terms of breadth, cross-reactivity and quality (avidity and pattern of cytokines secreted). These results are of relevance contributing to the design of strategies aimed to induce responses against HIV antigens with a superior breadth, avidity and cross-reactivity against multiple viral subtypes.

**Table 1:** Results summary.

	Prime:	DNA 3× NefBF plus	-	IL-12	GM-CSF	IL-12+GM-CSF
	Boost:		MVAnefBF	MVAnefBF	MVAnefBF	MVAnefBF
Magnitude	IFN-γ SFC/10^6^ cells median [range]		169 [79–296]	***457 [275–621]***	289 [142–477]	296 [176–845]
	Increase median respect to control group [range]		-	***2.38 [1.21–2.71]***	1.73 [1–1.91]	1.53 [1.05–2.86]
Breadth	Σ Frequency of NefBF structural domains recognized^a^		2	***3***	***3***	2
	Σ Frequency of PTE structural domains recognized		2	***2.33***	2	1
	Σ Frequency of BF peptides recognized		2	***2.32***	***2.32***	2
	Σ Frequency of B peptides recognized		1.08	***1.5***	***1.5***	0.66
Quality	Pattern of cytokines secreted^b^	IFN		+ + + + =	+ + + + =	+ + + − −
		TNF		+ + + = =	+ = = = =	+ + = = =
		IL-2		+ = = = =	− − = = =	+ + + + =
	Avidity	SD_50_ BF33 (µg/ml)	0.0676	0.0995	ND	ND
		SD_50_ B84 (µg/ml)	1.246	***0.4414***	ND	ND
	Prolif-erative capacity	%BF33-specific CD4^+^ cells	0.355	0.903	ND	***0.940***
		%BF33-specific CD8^+^ cells	0.02	1.395	ND	***1.855***

SD_50_: sensitizing dose of peptide concentration required to induce half-maximum of the maximum number of specific IFN-γ secreting cells.

ND: non determined.

a Sum of the recognition frequencies (from 0 to1) of each of the five NefBF structural domains. For example: *Control group: 1(N-t)+0 (Nt-C)+0(OCC)+1(L)+0(C-t) = 2*.

b +/−/ = : significant increase/decrease/or no-changes in the level of cytokines produced against each of the 5 targeted peptides compared to control group.

With bold and italic letter it is indicated the best response found compare to control group.

## Materials and Methods

### 1. Cell lines

Cell lines employed in the study were: BHK-21 (fibroblast cell-line derived from baby hamster kidney, ATCC Cat No CCL-10) and 293-T (epithelial cell line derived from human kidney cells, ATCC Cat No CRL-1573). Cells were maintained at 37°C in a 5% CO_2_ atmosphere in Dulbecco's Modified Eagle's Medium (Gibco BRL, USA) supplemented with 10% fetal bovine serum (Natocor), 2 mM Lglutamine (Gibco BRL), 100 U/ml penicillin (Gibco BRL) and 100 mg/ml streptomycin (Gibco BRL) (DMEMc).

### 2. Viral stocks for immunization

MVA recombinant virus encoding NefBF protein was previously developed in our lab [42]. MVAnefBF viral stock employed for immunizations were grown in BHK-21 cells. MVA stock was purified through 45% sucrose-cushion as elsewhere indicated [62] and then titrated by immunostaining of fixed BHK-21 infected cell-cultures with polyclonal serum reactive against VV proteins.

### 3. DNA for immunization

The DNA plasmid expressing NefBF was described before [42]. The DNA plasmids carrying the sequence coding for IL-12 [30], [39] and GM-CSF were kindly provided by Dr. Mariano Esteban, National Centre of Biotechnology of the Autonomous University of Madrid, Spain. In the case of GM-CSF vector, which has not been described previously, an assay to quantify the amount of cytokine released into the supernatant of transfected cells was conducted. For this, 293-T cells were transfected with 10 µg of DNA-GM-CSF, using Lipofectamine (Invitrogen, USA), following the manufacturer's instructions. The amount of cytokine was quantified in the supernatant by ELISA, 48 h post-transfection (ELISA Kit, Peprotech, Mexico). Transfected cells produced 150 µg/ml of GM-CSF in average (versus 0.2 µg/ml of untransfected cells) thus verifying the ability of the vector to express this molecule (*data not shown*).

Plasmids were purified with Endo free Maxi-Prep purification kits (NucleoBond Xtra Maxi Plus EF, Macherey-Nalgen, Germany) using pyrogen-free material and eluted in 200 µl/column pyrogen-free TE buffer and then diluted for injection in sterile PBS.

### 4. Mice immunization protocols, sample collection and processing

Six- to eight-week-old female specific-pathogen-free (SPF) BALB/c mice (H-2d) were purchased from the Laboratories of the School of Veterinary Sciences, University of La Plata, Buenos Aires, and then housed in our animal facilities. All experiments were done in compliance with legal and institutional guidelines [63]. The protocol was approved by the Committee of Care and Use of Laboratory Animals from the School of Medicine, University of Buenos Aires (Permit Number: 508/2009).

DNA doses (50 µg each one) were applied in 100 µl of sterile PBS by the intramuscular (i.m.) route, whereas immunizations with viral vectors (MVA, 10^7^ PFU) were given intraperitoneally (i.p.) in 200 µl PBS. In the immunization schemes applied, doses were always spaced by intervals of 14 days. Mice were sacrificed nine days after the last immunization and spleens were recovered under sterile conditions. Splenocytes were isolated by standard procedures. The number of animals of each of the experimental groups of the study was of 4. Then the T-cell assays were performed with pooled cells from the 4 mice of each group.

### 5. Peptides

Overlapping synthetic peptides (13–15-mers, overlapping by 11 amino acids (aa)) were designed based on the Nef protein from CRF12_BF reference strain ARMA185 (GenBank accession number AY037279), the same sequence employed for the construction of the DNA and MVA, and custom ordered from JPT Peptide Technologies (Germany) [20], [42]. They were used either as i) a single pool, representing the full protein; ii) as five pools, representing each protein domain: N-terminal domain (N-t, comprising since the first amino acid (aa) to 83), N-terminal Core (Nt-C, 84 to 115), Oligomerization domain and Central Core (OCC, 116–159), the Loop of the protein (L, 160–187) and the C-terminal domain (C-t, 188–205); iii) as 12 pools of 6 peptides generating a NefBF matrix where each peptide was represented in two different peptide pools, allowing for the identification of the respective peptide by responses in the two corresponding pools as previously described by Turk et al [20]; iv) or as single peptides.

Overlapping synthetic peptides of NefB consensus protein and PTE peptides were obtained from the NIH AIDS Reagent Program. The NefB peptides were used as i) a single pool, representing the full protein, ii) as 14 pools of 7 peptides forming a B matrix where each peptide was represented in two different peptide pools, allowing for the identification of the respective peptide by responses in the two corresponding pools as previously described [20], iii) or as single peptides.

The PTE peptide set represent the most frequent potential T-cell epitopes (PTE) embedded in the sequences of circulating HIV-1 strains of HIV-1 worldwide. The peptides are 15 aa in length. The global PTE peptides cover all PTEs with a frequency equal to or greater than 15% in any one of the subtypes A, B, C and non-ABC. Here, the PTE peptides were pooled in three pools representing the N-terminal (N-t, aa 1 to 83), Central (Cent, 84 to 159) and C-terminal (C-t, 160 to 205) portion of the protein.

In all the cases, lyophilized peptides were dissolved in dimethyl sulfoxide (DMSO) and stored at −20°C.

All the pools were used as stimulus in the ELISPOT assay, to stimulate the cells for subsequent ELISA assays and for flow cytometry assay. In all the cases the final peptide concentration was of 2 µg/ml.

### 6. Murine IFN-γ ELISPOT assays

ELISPOT assays were performed with freshly isolated splenocytes cultured in RPMIc (RPMI-1640 medium (Gibco BRL,) supplemented with 10% fetal bovine serum (Natocor), 2 mM Lglutamine (Gibco BRL), 100 U/ml penicillin (Gibco BRL), 100 mg/ml streptomycin (Gibco BRL), and 10 mM HEPES (Gibco BRL) and b-Mercaptoetanol….) as described previously [42]. Briefly, 0.125×10^6^ to 10^6^ cells were plated on duplicate or triplicate wells in 96-well ELISPOT plates (MultiScreen IP plates, Millipore, USA) previously coated with anti-mouse IFN-γ Ab (BD ELISPOT mouse IFN-γ ELISPOT pair). Peptide pools or single peptides were added at a final concentration of 2 µg/ml. Negative controls consisted of peptide-free medium plus 0.5% DMSO. Concanavalin A (ConA, Sigma-Aldrich, Germany) at 1 µg/ml was included as positive control. Plates were incubated for 36 h in a 37°C incubator with a 5% CO_2_ atmosphere, later washed extensively with PBS plus 0.05% Tween 20 (PBS-T) and incubated during 16–18 h at 4°C with the detecting Ab solution (BD ELISPOT mouse IFN-γ ELISPOT pair). Thereafter, plates were washed and incubated during 1 h with peroxidase-labeled streptavidin–avidin (BD PharMingen, USA). After 1 h, wells were washed and spots were developed by adding a 1 µg/ml solution of the substrate 3.3-diaminobenzidine tetrahydrochloride (Sigma-Aldrich) containing 0.015% hydrogen peroxide. Plates were scanned on an ImmunoSpot reader (Cellular Technology Ltd.). Specific spots were counted and the spot mean sizes were calculated using the ImmunoSpot software.

Functional avidity referred to as the activation threshold in response to defined concentrations of exogenous peptide was performed following the protocols previously described [46]. Briefly, limiting peptide dilutions (from 20 to 0.002 µg/ml) were performed and then the sensitizing dose of peptide concentration required to induce a half-maximum IFN-γ secreting cells (SD_50_) in *ex vivo* assays was determined with a sigmoid dose-response curve (GraphPad software).

### 7. T cell-specific cytokine production

Splenocytes were resuspended in RPMIc and cultured in triplicate wells (10^6^ cells/well) into 96-well microtiter flat-bottom plates and stimulated with single NefBF or NefB peptides at a final concentration of 2 µg/ml, Con A (1 µg/ml; Sigma–Aldrich), or medium alone with 0.5% DMSO. After 72 h of incubation at 37°C in 5% CO_2_, culture supernatants were harvested and stored at −80°C until analyzed by ELISA for IFN-γ TNF or IL-2 (BD PharMingen) following the manufacturer's instructions. Responses were considered positive when values obtained were higher than RPMI+2xSD.

### 8. Proliferation assay

Proliferation assay was performed using cryopreserved splenocytes. After obtaining splenocytes, remaining cells were cryopreserved in FBS plus 10% DMSO and stored in liquid N_2_ until their use. On day 1, cryopreserved splenocytes were thawed and rested during 6–8 hr in RPMIc, at 37°C in a 5% CO_2_ atmosphere. After resting, cell viability was checked by trypan blue exclusion.

To evaluate the proliferation of BF33-specific T-cells, splenocytes from mice were labeled with carboxyfluorescein succinimidyl ester (CFSE) [55]. Briefly, splenocytes were resuspended (10^6^ cells/ml) in PBS plus 1% FBS and labeled with 1.25 µM of CFSE (Molecular Probes, USA) at 37°C for 8 minutes. The reaction was quenched with FBS and cells were washed with RPMIc. Then, cells were resuspended in RPMIc at a density of 5×10^6^/ml. Cells were cultured in M12 plates for 5 days at 37°C and 5% CO_2_ atmosphere with medium only or 2 µg/ml of BF33 peptide. Positive controls were stimulated with ConA. At the fifth day, splenocytes were harvested, counted and washed with RPMIc. Then, cells were stained for 20 minutes at 4°C with anti-mouse CD3 allophycocyanin (APC), peridinin chlorophyll protein (PerCP)-conjugated anti-mouse CD4 or CD8 monoclonal antibodies (BD Biosciences) plus viability dye near-IR-fluorescent reactive (LIVE/DEAD, Invitrogen). Cells were then washed and acquired in a BD FACSCanto flow cytometer. Data acquisition and analysis was performed using the BD FACSDiva software. Instrument setting and fluorescence compensation were performed for each day of testing using unstained and single-stained samples. Initial gating was performed on small lymphocytes in a forward scatter versus side scatter (SSC) plot. At least 100.000 events were acquired in the lymphocyte gate. The viable cells were gated on a LIVE/DEAD histogram. The percentage of CD3^+^CD4^+^ and CD3^+^CD8^+^ proliferating (CFSE low) cells was determined after gating cells in a CD3 versus CD4 or CD8 plot.

### 9. Bioinformatic analysis

The MHC binding affinity of peptides was predicted using the web based immunology tools SYFPEITHI Epitope Prediction (Class I: Ld and Kd; Class II: Ad and Ed) and PREDEP (Class I: Dd) prediction softwares.

### 10. Data analysis

In ELISPOT and ELISA assays, positivity threshold in peptide-stimulated wells was calculated as at least 2 times the average plus one SD value of negative control wells for each group.

Mann-Whitney test was used to compare inter-group responses in figure 1 (n>3). Student's t-test was used to compare frequencies among the groups. For all tests, a value of p<0.05 was considered statistically significant.

## Supporting Information

Figure S1 **IL-12 and GM-CSF enhanced the NefBF IL-2 immune response.** Nine days after the boost dose, cellular immune response against NefBF was evaluated in the spleen, quantifying the number of specific IL-2 secreting cells by ELISPOT. Background values found in negative control wells (RPMI plus DMSO) were subtracted and ranged from 5 to 30 spots/well. Bars represent the average of duplicated samples plus SD. Data is from a representative experiment out of two. Significant differences (T test) between control vs the other three groups are indicated with * p<0.05 and **p<0.005.
(TIF)
