# Supplementary figures and images for: IL-12 and GM-CSF in DNA/MVA Immunizations against HIV-1 CRF12_BF Nef Induced T-Cell Responses With an Enhanced Magnitude, Breadth and Quality
Source: PLoS One. 2012 May 24;7(5):e37801. doi: 10.1371/journal.pone.0037801 (PMC3360004; doi:10.1371/journal.pone.0037801)

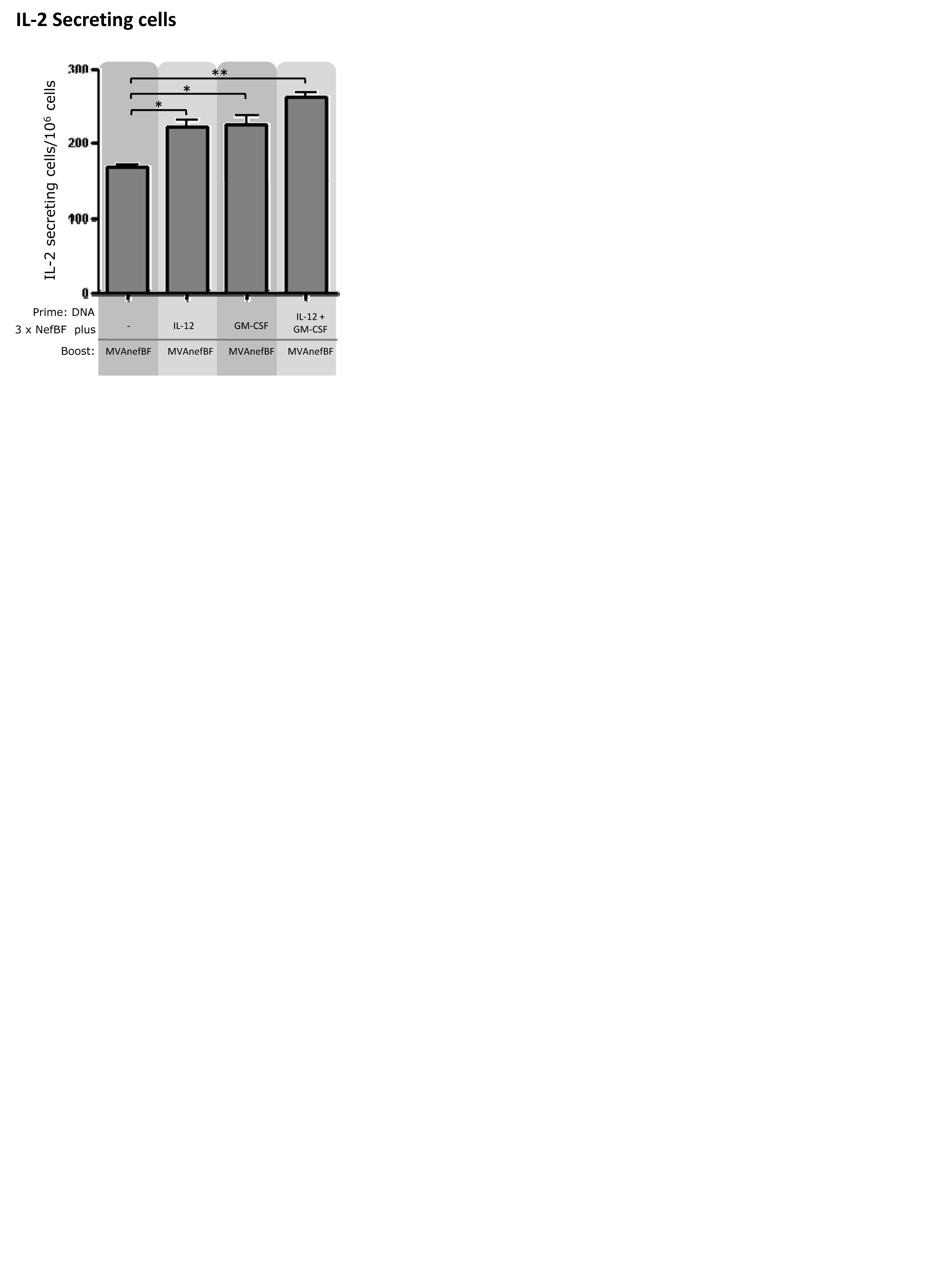

Supplement: Figure S1 — IL-12 and GM-CSF enhanced the NefBF IL-2 immune response. Nine days after the boost dose, cellular immune response against NefBF was evaluated in the spleen, quantifying the number of specific IL-2 secreting cells by ELISPOT. Background values found in negative control wells (RPMI plus DMSO) were subtracted and ranged from 5 to 30 spots/well. Bars represent the average of duplicated samples plus SD. Data is from a representative experiment out of two. Significant differences (T test) between control vs the other three groups are indicated with * p<0.05 and **p<0.005. (TIF) [file pone.0037801.s001.tif]
